# The Association of Microbiome Dysbiosis With Colorectal Cancer

**DOI:** 10.7759/cureus.22156

**Published:** 2022-02-12

**Authors:** Artem Artemev, Sheetal Naik, Anastasia Pougno, Prasanna Honnavar, Nandan M Shanbhag

**Affiliations:** 1 Medicine, Xavier University School of Medicine, Oranjestad, ABW; 2 Physiology, American University of Antigua, St. Johns, ATG; 3 Microbiology and Immunology, American University of Antigua, St. Johns, ATG; 4 Oncology, Neuro Spinal Hospital, Dubai, ARE

**Keywords:** cancer genomics, colon cancer prevention, microbiome, bacterial dysbiosis, gut microbiota, colorectal cancer

## Abstract

Many studies have been conducted to identify the causative organisms in colorectal cancer (CRC) and compare the microbiota of healthy individuals and those with CRC. The pathways by which the microbiota promotes CRC development are not yet fully understood. The hypothesized mechanisms include damage to the DNA, production of carcinogenic metabolites, and promotion of chronic inflammation. In a state of dysbiosis, the gut loses protective bacteria and is enriched with pathogenic and cancer-promoting bacteria, which promotes functions associated with cancer such as angiogenesis, loss of apoptosis, and cell proliferation. We have established a strong link between microbiota dysbiosis and certain species of bacteria and even viruses involved in tumorigenesis. In this review, we look at some of the major identified species and how they are related to CRC. Future research should include and even focus on mycobiome and virome on CRC development. Due to the diversity of the gut microbiome, there is a high possibility that the gain and loss of bacteria and their metabolic functions lead to CRC.

## Introduction and background

Colorectal cancer

Cancer is characterized by the loss of cell function and its regulation and cellular division in bodily fluids and certain tissues. Uncontrollable cellular growth has the capacity to invade adjacent tissues. These invasive tumors are termed “malignant” [1]. Colorectal cancer (CRC) is the third most common malignancy in western countries [2]. The United States has an estimated 135,430 new cases of CRC, followed by over 50,000 deaths annually [3]. There are 600,000 CRC-related deaths annually in the whole world, translating to 8% of the total cancer deaths worldwide [4]. In the United States, CRC is 8.6% of all new cancers diagnosed [5]. CRC is seen mostly in older adults and peaks around the sixth decade of life [6]. Less than 20% of CRCs are found in individuals younger than the age of 50, and in the USA, less than 1% of CRC are seen in the first 20 years of life; there is an increase in CRC in the young population. These findings raise the question of lifestyle risk factors and even infectious etiology. The American Cancer Society found an increase in the incidence of CRC of 2.9% per year in women and 3.5% in men from 1992 to 2005, worldwide. In contrast, there is a decline of 20% in CRC incidences in the population over the age of 50. Overall, an individual has a CRC lifetime risk of about 5% [7].

CRC includes adenocarcinomas (ADCs), adenosquamous carcinoma, squamous cell carcinoma, undifferentiated carcinoma, signet cell ring carcinoma, and spindle cell carcinoma [8]. Each histological type has different clinical features and requires slightly different management and treatment. The main sporadic form of CRC is common in older individuals. First, it develops from a pre-existing polyp, leading to an adenoma-dysplasia-carcinoma sequence, and progresses to malignancy over a period of five to 10 years [4]. This slow progression makes CRC one of the most preventable cancers, especially since it arises from benign neoplasm, which allows early detection and removal of dangerous polyps before they undergo malignant transformation [8-9]. The polyp to CRC sequence has multiple possible ways to the development of a cancerous tumor [9]. CRC usually originates from epithelial cells, which replicate at a high rate, which poses risks of mutation and carcinogenesis to the site when defensive mechanisms fail due to many factors [8]. However, the small intestine is much less vulnerable to such risks despite having a high cell turnover rate as well [10].

CRC risk factors

Human CRC can be inherited, inflammatory due to diseases such as Crohn’s and ulcerative colitis, and sporadic, which accounts for over 80% of CRC with a poorly understood etiology [11]. Risk factors for CRC include advanced age, high body mass index (BMI), consumption of alcohol and processed and red meats, and chronic inﬂammation of the gastrointestinal tract due to inflammatory disorders [12-13]. The role of genetic alterations in CRC was first reported by Fearon and Vogelstein [14]. The key players in CRC are mutations in KRAS, tumor suppressor genes (APC), CTNNB10, and p53, as well as modifications in pathways leading to mismatch repair (MMR), chromosomal and microsatellite instability (MSI), and hypermethylation of promoter CpG island sites (CIMP) [14]. Due to such mysterious etiology to 80% of CRCs, it has been proposed to add microbial dysbiosis as a risk factor and study such a hypothesis. This can be attributed to environmental exposures, such as infections, antibiotics, probiotics, and diet. It is hypothesized that gut microbiota in association with processed food diet is a risk factor for CRC [12]. The gut microbiome has been linked to inﬂammatory bowel disease and obesity [13]. However, the knowledge of the composition of the microbiota developing CRC is sparse [12]. This paper explores the main organisms that contribute to CRCs development and the protectives and harmful roles of such organisms.

Gut microbiome

We have a symbiotic relationship with our microbiome. We provide a nutritious habitat, and the bacteria develop the immune system, maintain the physiological environment, provide nutrients, prevent colonization by pathogenic bacteria, and even metabolize procarcinogens and carcinogens [11,15]. The bacteria also aid in the absorption of carbohydrates, and the production of crucial vitamins B and K [14]. The health of the digestive system and the colon very much depend on the contribution of the microbiome [16]. The human intestine allows the habitation of more than 1000 different species of bacteria, mostly in the colon, and overall over 100 trillion gut microbial cells [10,16]. The microbiota composition and proportion depend on oxygen and nutrients availability, pH, diet, among other factors. It also differs across the lumen, mucosa, and crypt-villus axis. Sixty percent (60%) of the composition is Firmicutes, 20% is Bacteroides, followed by Actinobacterium and Enterobacteriaceae, as well as viruses, archaea, and fungi [14,16]. It is thought that certain individuals carry different proportions of bacteria in the system due to genetic makeup, lifestyle, and environment, which could predispose them to obesity, autoimmune disorders, inflammatory diseases, and even cancers [15]. The bacterial concentration in the large intestine and in the small intestine is in the ratio of 1012 to 102 cells /mL. CRC risk in the large intestine is 12-fold. This allows the hypothesis that some of CRC etiology is due to bacteria. Roughly 16% of all cancers are caused by microbes, and some gastrointestinal tract and liver cancers are clearly identified to be triggered by microbes [17]. Although the gut microbiome is a constant topic of clinical and lab studies and is widely discussed in the literature, one emerging and growing area of the gut microbiome is its role in cancer pathogenesis. Due to the great number of microbes in the gastrointestinal tract and a growing body of research linking infectious agents as a cancer cause, researchers have started looking closely at the link between the gut microbiome and infectious agents and CRC [10]. Multiple studies show that microorganisms are potential contributors to CRCs through the toxic metabolites produced, immune system interaction, and the release of genotoxic virulence factors [3].

## Review

How does the microbiota lead to CRC?

The first-known time that clinicians and researchers looked into the gut microbiota, linking it to CRC was in 1971, in a study that identified 13 bacterial species associated with CRC. The study showed the Bacteroides family as principal in patients with CRC. Bacteroides fragilis is an intestinal commensal and shown to induce spontaneous colonic tumorigenesis in mice [15]. Although premature, the study did open doors for more studies and research, advancing our knowledge of CRC etiology. The pathways by which the microbiota promotes CRC development are not yet fully understood. Hypothesized mechanisms include damage to DNA, production of carcinogenic metabolites, and promotion of chronic inflammation [14]. In a state of dysbiosis, the gut loses protective bacteria and is enriched with pathogenic and cancer-promoting bacteria, which promote functions associated with cancer such as angiogenesis, loss of apoptosis, and cell proliferation [8,18]. Therefore, tumor development in the colon is affected by the configuration of the microbiome. Evidence shows that E. coli and B. fragilis can directly affect tumor growth in the colon, whereas bacteria that produce the short-chain fatty acid (SCFA) butyrate have anti-tumor properties, thus proving that the microbiota has both harmful and protective functions. It has been shown that antibiotics manipulate the microbiota and reduce tumor formation, proving the importance of bacteria as drivers of tumorigenesis [18]. When microbiota from mice with tumors was transplanted into sterile mice, it amplified the amount and size of the cancerous tumors developed, as opposed to the mice transplanted with healthy microbiota from healthy mice [13].

There are a few mechanisms by which microorganisms are linked to CRC development. One of the mechanisms is the initiation of inflammation [8,14]. Inﬂammatory bowel diseases have been directly linked to CRC development. Chronic inﬂammation leads to reactive oxygen species and inﬂammatory cytokines that create a microenvironment that helps carcinogenesis [13,18]. It is a possibility that microbiota dysbiosis is caused by inflammation, as much as that dysbiosis results in inflammation. Communications between the CRC and intestinal microbiota may go either way, where the inflammation and tumor promote dysbiosis, or a dysbiotic microbiome promotes carcinogenesis. Also, a host could actively “select” microorganisms by having a particular receptor recognized by a pathogenic organism. A host could passively “select” microorganisms by having an environment favorable to the growth of select pathogenic microorganisms. These “selections” could tip the balance towards the development of inflammation [15].

In the past, scientists assumed that stress and altered microbiota trigger inflammatory diseases in the intestines [18]. A recent discovery by scientists from Munich showed that tumor growth in the colon can also be triggered by a combination of altered gut microbiota and cell stress [16]. They showed that in the intestine of germ-free animals, activated transcription factor 6 (ATF6) regulated stress. In contrast, when the microbiota was transplanted into these animals, tumors developed in the colon. Indeed, ATF6 incidence is increased in many colon cancer patients, with close to 11% of CRC patients showing an increase in ATF6 [19]. As shown by data from 541 patients with CRC, those whose ATF6 was increased were at a higher risk of recurrence, and 10% of these patients had a greater risk of getting CRC again. Thus, it is proposed to use ATF6 as a diagnostic marker for CRC [20].

Another mechanism by which microbiota drives carcinogenesis is metabolic and hormonal changes. Changing from a low to a high-fat diet abruptly is associated with a change in the balance of the phyla, Bacteroidetes and Firmicutes, due to the increase in the Firmicutes. Comparable alterations in the proportion are seen in overweight and obese humans [15]. Studies revealed that obese mice are more likely to develop preneoplastic colonic changes than non-obese mice due to insulin-like growth factors that trigger mitosis control cell proliferation and inhibit apoptosis. Thus, overweight and obese individuals who also suffer from diabetes are at a high risk of developing CRC due to these factors.

A promising theory is that commensal bacteria are a trigger for polarizing colonic macrophages, linking the microbiota to COX-2 and inflammation. A novel mechanism of inducing the chromosomal instability (CIN) pathway in colonic cells is called the “macrophage-induced bystander effect" (BSE). In this mechanism, commensals polarize macrophage to an M1 phenotype that results in mutation and other pathways resulting in malignant alterations [21].

It is hypothesized that bacteria can cause damage to the DNA and although it is not well-established, we know now that enteropathogenic Escherichia (E.) coli can induce tumorigenesis by downregulation of DNA mismatch repair [15]. Stress, diet, and environmental toxins are also thought to be able to influence the microbiota and result in failure to prevent epithelium renewal when stem cells’ genes are altered.

Association of microbiota in CRC

The gut microbiota mainly consists of Bacteroidetes and Firmicutes. Predominant genera include Eubacterium, Bacteroides, Ruminococcus, Bifidobacterium, Propionibacterium, Peptostreptococcus, Lactobacillus, Clostridium, Streptococcus, and Escherichia [22-23]. Many studies have been conducted to identify the causative organisms in CRC and compare the microbiota of healthy individuals and those with CRCs. 

While Streptococcus bovis was considered to be involved in endocarditis, its association with CRC was recognized in 1974, sparking interest in the microorganisms’ link to CRC [15]. The majority of the early conducted studies in the field confirmed a dysbiosis in CRC tumors as compared with healthy mucosa in healthy individuals [22]. This sparks a debate on whether malignancy results in dysbiosis or the dysbiosis in the gut result in tumorigenesis. One study found an increase in gut adherent microbes, such as Ruminococcus obeum and Allobaculum spp. in precancerous lesions, suggesting such microbes play a role in adenoma development [14].

An extensive study in the field by Gao et al. found a dysbiosis in CRC patients and identified the over-representation of Firmicutes, Fusobacteria, and Lactococcus, with under-representation of Proteobacteria, Escherichia, Shigella, and Pseudomonas in cancerous tissues as compared to healthy surrounding tissues in the same patients. When comparing tumor tissue and healthy adjacent tissue, Firmicutes comprised 63.46% and 39.54%, respectively, and Bacteroidetes 12.77% and 19.1%, respectively. On the corollary, Proteobacteria was three times higher in healthy tissue at 36% versus 10.66% in the tumor tissue [24]. The same study also found that more species were found in cancerous tissues than in healthy tissues, dominated by Firmicutes, Bacteroidetes, and Proteobacteria, which account for over 98% of the phylum [24]. When comparing distal and proximal tumors among CRC patients, the distal colorectal tumors had more of Fusobacterium and Escherichia-Shigella, while in the proximal tumors, there was a greater abundance of Peptostreptoccus, Selenomonas, Prevotella, and Pyramidobacter [22]. Another extensive study by Hibberd et al. revealed the difference in gut microbiome between patients with and without CRC. There was an increase in the diversity of the microbiome in CRC patients, and it concluded that microbiota is a risk factor for CRC [12]. Interestingly, the researchers discovered that the tissue microbiota of patients with CRC was more similar within biopsies than among healthy individuals, which suggests that healthy microbiota composition may vary while tumor-associated microbiota has to be constant to promote tumorigenesis or is a result of the CRC. The tumor microbiota was rich in taxa, such as Fusobacterium, Selenomonas, and Peptostreptococcus, while Peptostreptococcus dominated in mucosal and fecal samples. Among biopsies taken from CRC patients’ tumors, adjacent mucosa, and fecal matter, there was a consistent overabundance in other species as well, including the phylum Euryarchaeota, genus Methanobrevibacter, and from within Firmicutes, Peptostreptococcus, Oscillospira, Selenomonas, Rikenellaceae spp (phylum Bacteroidetes), and Bilophila (phylum Proteobacteria). Fusobacterium tended to co-occur with Peptostreptococcus, Bulleidia, and campylobacter. Streptococcus was depleted in these samples. The CRC-associated microbiota had an increased abundance of Fusobacterium and Peptostreptococcus, with both genera containing opportunistic oral pathogens with a high capacity to colonize mucosa and form a biofilm, which suggests similar colonization of the intestines [12,22]. Multiple studies show that F. nucleatum, P. Micra and P. anaerobius have an increased abundance of 132-fold, 41-fold, and 37-fold, respectively, among CRC patients, and could be promising tumor markers.

A different study shows that gram-negative bacteria, such as Bacteroides, Parabacteroides, Alistipes, and Akkermansia, are associated with CRC. Indeed, Bacteroides and Akkermansia break down glycans, specifically mucin, resulting in inflammation and colonization of intestines by pathogens. In contrast, gram-positive bacteria, such as the Clostridiales family, are inversely correlated with tumors [25].

Bacterial drivers may not be detected in tumors, as they are replaced by passenger bacteria that grow in the rich tumor environment. Species such as Citrobacter, Shigella, Bacteroides, and Salmonella are drivers due to their abundance in the early stages of CRC, and later disappear as the diseases evolve. Streptococcus gallolyticus, Fusobacterium spp., Clostridium septicum, and Coriobacteriaceae are considered passenger bacteria, as they are found in advanced tumors [12,15,22].

MicroRNAs (miRNAs) have altered expression profiles in CRC. One study found that miRNA vastly differ in their expression between tumor and normal tissue. Small RNAs from F. nucleatum and Epstein-Barr virus (EBV) are greater in tumors than in adjacent normal tissue. Other viruses, such as Human Cytomegalovirus and John Cunningham virus, have been detected in CRC tissue [22,26].

We have established a strong link between microbiota dysbiosis and certain species of bacteria and even viruses involved in tumorigenesis. We will now take a better look at some of the major identified species and identify how they are related to CRCs.

Bacteria

Streptococcus bovis 

Streptococcus (S.) bovis is the normal flora of the gut in up to 16% of adults. Proteins released from S. bovis stimulate overexpression of COX-2 and inflammation, which is more often found in CRCs and can hinder apoptosis and upsurge angiogenesis. The S. bovis association with CRC was described as early as 1966 [3]. Studies reported that neoplasia of the colon was observed in 6% to 71% of S. bovis bacteremia cases, thus colonoscopy has been recommended in this group. One study found that 55% of S. bovis endocarditis cases had colorectal neoplasia. S. bovis possesses a cell wall antigen that is attracted to the collagen IV in the colon mucosa, and it also induces the production of pro-inflammatory cytokines interleukin (IL)-8, IL-1, and COX-2, which, in turn, result in angiogenesis and cell proliferation while decreasing malignant cells’ apoptosis. Streptococcus gallolyticus (S. bovis biotype I) was seen in 71% of CRC compared to other S. bovis subtypes, which had an incidence of 17%. Forty-nine percent (49%) of CRC tumor specimens were in the DNA of S. gallolyticus compared to 8% of healthy tissues. Higher rates of CRC were linked to the presence of S. gallolyticus IgG antibodies. It showed that S. gallolyticus serum antibodies were positive in 68% of CRC patients, 78% of adenoma patients, and only 17% of control patients [3,10]. 

Sulfidogenic Bacteria 

Sulfidogenic bacteria like Desulfovibrio, Fusobacterium, and Bilophila wadsworthia, through the production of H2S, have been accused of CRC development. H2S is a genotoxin that was found in over 80% of sporadic CRCs cases and is known to damage DNA leading to genomic instability and a high incidence of mutations. H2S can hinder mitochondrial function resulting in cell hyperproduction through the Ras/MAPK pathway, a known mechanism of carcinogenesis. A diet high in meat and fat correlates positively with a high concentration of sulfidogenic bacteria [3,22].

Fusobacterium nucleatum is an anaerobic Gram-negative bacillus that has recently been the center of attention after researchers noted its link to CRC. It commonly inhabits the oral cavity, occasionally causing periodontal and gingival infections [27]. Patients with CRC have a greater concentration of F. nucleatum compared to healthy individuals. The higher tissue concentrations of F. nucleatum have been linked to later stages of CRC and lymph node involvement [28]. One study showed that lymph node metastases were evident in 59% of patients with an abundance of F. nucleatum and in no patients with low concentrations of F. nucleatum [3,22]. F. nucleatum is also associated with survival rates. Studies have attributed the high concentration of F. nucleatum to a low-fiber and high-fat diet. F. nucleatum produces H2S, which attaches to the cell’s surface and enters epithelial cells through its surface virulence factor, FadA, by binding to E-cadherin, generating proinflammatory cytokines. Although normally, E-cadherin is a tumor suppressor, by binding with FadA, its tumor suppressor activity is inhibited. In addition, FadA and E-cadherin attachment leads to the activation of β-catenin signaling to start inflammatory and pro-oncogenic pathways, resulting in tumorigenesis. Despite the link between F. nucleatum and CRC, it is still unknown whether Fn colonization is a consequence or a cause of CRC [27].

Bacteroides fragilis 

**Figure 1:**
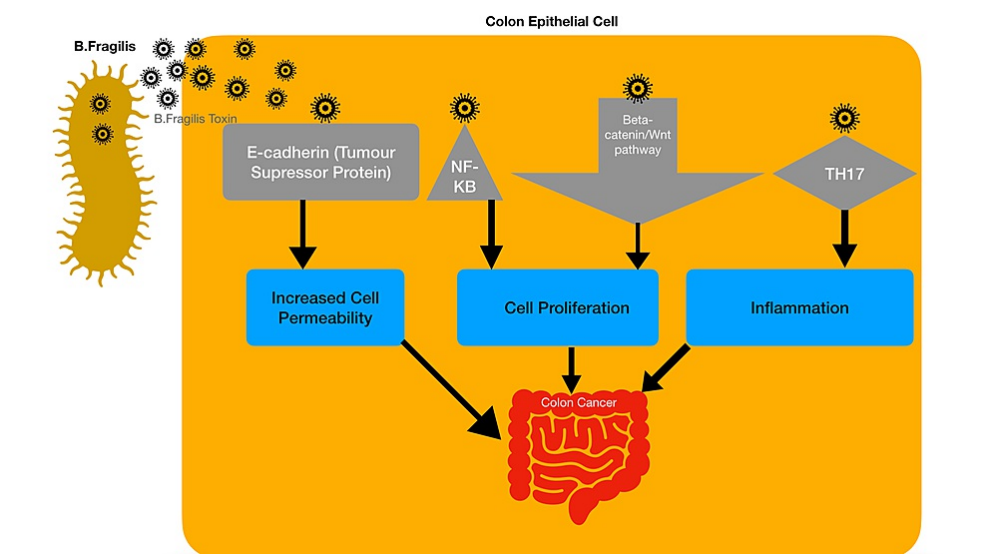
Pathophysiology of Bacteroides fragilis toxin resulting in colorectal cancer

Bacteroides (B.) fragilis is an anaerobic bacterium and is very common in the human body. The Enterotoxigenic Bacteroides fragilis (ETBF) strain has the B. fragilis toxin (BFT) [3]. The pathophysiology of BFT resulting in colorectal cancer has been recognized (Figure 1).

BFT activates the Wnt/β-catenin pathway to increase cell proliferation. It also activates NFkB to promote inflammatory mediators, leading to inflammation and carcinogenesis [14,22]. ETBF attaches to colonic epithelial cells and stimulates cleavage of the tumor-suppressor E-cadherin, resulting in increased permeability of the cell. Research also established that ETBF promotes carcinogenesis by the T helper 17 (TH17) dependent pathway. Toprak et al. found B. fragilis in the stool specimens of 56 out of 73 CRC patients, among which B. fragilis was found in 21 with the BFT gene. This is abundant when compared with ﬁve of the 40 healthy controls [29]. In a study by Boleij et al., findings show that the mucosa of cases was signiﬁcantly more often BFT-positive compared with control biopsies. There was 100% BFT positivity in tissues from late-stage CRC versus 72.7% positivity in early-stage CRC [30]. B. fragilis abundance in CRC mucosa is a predictor of three-year overall survival [3,22].

Clostridium septicum 

Clostridium (C.) septicum is an obligate gram-positive anaerobic bacillus. This normal flora can cause bacteremia and gas gangrene (myonecrosis), which can be highly fatal [3,22]. Colon malignancy is seen in 71-85% of patients with C. septicum gas gangrene. C. septicum flourishes in necrotic, hypoxic, and acidic tumors. The alpha-toxin released by C. septicum induces necrosis, resulting in mucosal ulceration and invasion of bodily fluids. C. septicum has been linked to the development of malignancies although it has not been linked to carcinogenesis. Given the facts, CRC screening is recommended after the diagnosis of C. septicum myonecrosis or bacteremia.

Escherichia coli 

Escherichia (E.) coli is the predominant species of the normal intestinal microbiota and is aero-anaerobic Gram-negative bacteria [22,31]. E. coli is composed of the A, B1, B2, and D groups. Among E. coli virulence factors, cyclomodulins (CM) moderate cellular apoptosis, differentiation, and proliferation. It has been observed that the biopsies of patients with CRC have a higher prevalence of B2 phylogroup (55.3%) [18,31]. Compared to healthy individuals, adenocarcinoma patients have more mucosa-associated E. coli. Bacteria were present in biopsies from 92% of patients with CRC. Seventy percent (70%) of patients and only 3% healthy controls had the E. coli Polyketide synthase (PKS) genotoxic island of genotoxic E. coli strains, which may promote DNA double-strand breaks resulting in CRC14 and in G2/M cycle arrest [21].

Helicobacter pylori 

Helicobacter (H.) pylori is a Gram-negative bacteria associated with gastric pathologies and cancers. However, the association between H. pylori and CRC is not yet clear. As H. pylori promotes oxidative stress, there may be a higher possibility of CRC in H. pylori-infected individuals. One study reports an association between CagA seropositivity and an amplified risk in colonic cancer. Another study reports that there is a 10.6-fold increased risk of CRC in CagA+ H. pylori strains compared to CagA- strains [3]. H. pylori CagA+ strains can cause inflammation and malignancy [10]. There are many hypotheses put forward to show the link between H. pylori and carcinogenesis. One hypothesis states that CagA+ strains increase the serum levels of gastrin, resulting in hypergastrinemia, by its direct action on gastrin-secreting G cells, which may proliferate intestinal epithelium. Another mechanism is by decreasing the activity of somatostatin-secreting D cells that would otherwise inhibit the activity of G cells during high acid production, thus indirectly increasing serum gastrin levels. One study has linked hypergastrinemia to colorectal neoplasia.

Enterococcus faecalis 

Enterococcus (E.) faecalis can induce COX-2 by polarizing colon macrophages, resulting in the production of BSE, which results in CIN and mutations [21]. Experiments show that certain strains of E. faecalis promote extracellular superoxide release, which is then converted by H2S and could induce CIN, damaging the DNA, and carcinogenesis [14].

Acidovorax spp.

Acidovorax spp. is a member of the phylum Proteobacteria. It is linked with great risk for CRC. It metabolizes nitro-aromatic compounds and flagellar proteins that induce local inflammation [14].

Virome

Human papillomavirus (HPV)

HPV invades epithelial cells via microscopic tears. There are over 100 types of HPV, with types 16 and 18 the most cervical cancer-prone. A study by Buyru et al. found that CRC due to HPV 16 and HPV 18 diagnosed in 51.8% of cases. Evidence showed codon 12 mutations in 31 cases out of 53, with the HPV genome found in 43 out of 53 cases, and no K-ras mutations were found in normal controls. Fifty-five percent (55%) of HPV-positive samples had codon 12 mutations in K-ras. HPV 18 was found in 73.6% of samples and HPV 33 was found in 56.6% of the samples. K-ras protooncogene, once activated by mutation in codons 12 and 13, is unable to attach to the GTPase activating protein, keeping an activated state, promoting cancer development. K-ras mutations are involved in up to 50% of sporadic CRC [32]. Expression of HPV proteins E6 and E7 inhibits tumor suppressor proteins p53 and pRb resulting in the development of CRC. In order to develop CRC, other factors capable of provoking the disease need to take place. HPV presence alone does not cause tumorigenesis and requires alterations in the K-ras and p53 genes. Many studies have proven HPV 16 and 18 to be high-risk factors in the development of CRC, and other studies have shown K-ras mutation is common in CRC [32-33]. Through the addition of HPV DNA into human DNA, inactivation of tumor suppression genes, and expression of oncoproteins, HPV promotes oncogenesis [34].

John Cunningham virus (JCV)

JCV infection is extremely common, especially during early childhood, affecting up to 80% of the population, with the majority of the infected people being asymptomatic, and the virus remains latent in the kidneys [17]. A study by Burnett-Hartman found that JCV DNA was present in both normal and cancerous tissue, however, the JCV DNA number was significantly higher in the cancerous cells. JVC's large T-antigen protein attaches p53 and pRb, blocking tumor suppression and immortalizing cells in culture. Another protein, the agnoprotein, could also inhibit genomic instability mechanisms and cell cycle control. T-Ag, through greater migration and invasion, could facilitate metastasis in CRC cells. Various studies detected JCV DNA in CRCs, finding up to 89% of carcinomas positive for JCV. One study showed that out of 19 CRC patients. four had a JC virus-positive polymerase chain reaction (PCR) test [17].

EBV

Studies have found EBV viral DNA in CRC tumors. EBV proteins may affect the replication of host cells via viral miRNAs. miR-BART19-3p (miRNAs) is shown to attack WIF1 (host mRNAs), which is an important colon cancer gene, while miR-BART1 (miRNAs) attacks PSAT1 (host mRNAs), a tumor cell promoting gene [26].

Mycobiome

The fungal microbiome, known as Mycobiome, is a vital part of the gut microbiota although it is much less researched and discussed than bacteria or viruses. The most commonly found genera in a healthy gut are Candida (C.), Saccharomyces, and Cladosporium. Some foreign fungi from the diet or environment can also be found in the gut. These foreign fungi may comprise possibly pathogenic species. The gut was found to be colonized by the oral microbiome [34]. There were identical C. albicans strains in the mouth as well as colon of IBD patients. One study on fungal microbiota in CRC compared the microbiota in adenomas and adjacent tissue. There was an abundance of Candida and Phoma genera and adenomas had C. tropicalis [31].

Despite the seemingly low importance of the mycobiome, new studies have shown its importance in the gut. A study by Coker et al. revealed CRC-associated altered fungal composition. In both the case and control groups, the phylum Ascomycota and Basidiomycota dominated the mycobiota. However, in CRC, the ratio of Basidiomycota to Ascomycota is higher, with a decrease in Saccharomycetes and Pneumocystidomycetes and an increase in Malasseziomycetes. The study opens the door to further research into the importance of the mycobiome and fungal composition in CRC [35].

Parasites

Among CRC histological types, squamous cell cancer (SCC) is very rare, and its risk factors include Schistosomiasis and Amoebiasis. Studies from Japan and China, between 1983 and 2005, found a positive and signiﬁcant correlation between CRCs and mortality due to schistosomiasis. Schistosomal eggs were predominantly found in CRC patients than healthy individuals or those with benign tumors [36]. There has been a long debate on the correlation between Schistosoma (S.) japonicum and CRC, with the first report dating back to the 1950s. Since then, many reports from Japan and other parts of Asia have shown a correlation between CRC and evidence of infection by S. japonicum. Schistosomal colitis has been found to be associated with the early onset of CRC by targeting oncogenes. S. mekongi has been seen in leiomyosarcoma of the small intestines while S. intercalatum infection has been found in those with rectosigmoid carcinoma. Yet, the two species have not been confirmed as causative agents [37]. Other than the correlation between gastrointestinal neoplasia and schistosome species there is no other evidence. More research in the matter is therefore required. The major probable causative organisms have been studied and a summary is provided (Table 1).

**Table 1:** The main known culprits of CRC and their respective pathogenesis CRC: colorectal cancer

S. No	Causative organism	Pathogenesis in CRC	References
1	Streptococcus bovis	S. bovis possesses a cell wall antigen that is attracted to the collagen IV in the colon mucosa, and it also induces the production of pro-inflammatory cytokines IL-8, IL-1, and COX-2 overexpression, which, in turn, results in angiogenesis and cell proliferation while decreasing malignant cells’ apoptosis.	10
2	Sulfidogenic bacteria	H_2_S, after invading the epithelial cells, hinders mitochondrial function, resulting in cell hyperproduction by the Ras/MAPK pathway.	3
3	Fusobacterium nucleatum	Produce H_2_S, invades the host cell, generating proinflammatory cytokines through its surface virulence factor, FadA, by binding to E-cadherin. Although E-cadherin is a tumor suppressor, its tumor suppressor activity is inhibited by binding with FadA. Also, the binding of the FadA adhesion molecule and E-cadherin results in activating the β-catenin signaling pathway, inducing pro-oncogenic pathways, also leading to tumorigenesis.	3
4	Bacteroides fragilis	BFT activates the Wnt/β-catenin signaling pathway to cell proliferation, as well as stimulates NFkB, inducing inflammatory mediators, leading to inflammation and carcinogenesis. Secretion of BFT stimulates cleavage of the E-cadherin (tumor-suppressor protein), leading to cell permeability. In certain CRCs, E-cadherin stimulation magnifies cell signaling via the β-catenin/Wnt pathway.	3, 13-14, 22
5	Clostridium septicum	Its alpha-toxin induces necrosis resulting in mucosal ulceration and spread.	3, 22
6	Escherichia coli	Of the virulence factors, cyclomodulins (CM) moderate apoptosis, cellular differentiation, and proliferation. Genotoxic E. coli strains damage DNA. E. coli having the polyketide synthase (PKS), initiates the DNA double-strand breaks, promotes CRC, and brings about G2/M cycle arrest.	14, 18, 21, 31
7	Helicobacter Pylori	CagA+ strains are responsible for malignancy. Gastric H. pylori by directly stimulating G cells increases gastrin serum levels, or indirectly by inhibiting somatostatin producing D cells, leading to hypergastrinemia. This may have proliferative effects on the intestinal mucosa.	10
8	Enterococcus faecalis	Can polarize colon macrophages and thus induce COX-2 to produce BSE, resulting in mutations and CIN. Certain strains of E. faecalis promote extracellular superoxide release, which is converted by H_2_O_2_ and could damage DNA damage, CIN, and cancer.	14, 21
9	Acidovorax spp	Induces inflammation via its flagellar proteins and induces amplified metabolism of nitro-aromatic compounds.	14
10	Human papillomavirus	HPV expresses proteins E6 and E7, which inhibit p53 and pRb tumor suppressor proteins, leading to malignancy. HPV DNA is transported by exosomes to normal and neoplastic cells. By integrating the HPV DNA into the human DNA, HPV expresses oncoproteins.	10, 33
11	John Cunningham Virus	Its viral protein, large T-antigen, can immortalize cells in culture. The large T-antigen blocks tumor suppression and induces uncontrolled cellular replication by binding to p53 and the pRb family of proteins, resulting in chromosomal instability. Another protein, the agnoprotein, hinders cell cycle control.	10, 17
12	Epstein Barr Virus	EBV miRNAs attack host mRNAs and some target carcinogenesis genes. miR-BART19-3p targets WIF1, a gene important in CRC. miR-BART1 targets PSAT1, a gene promoting the replication and proliferation of tumor cells.	26

Anti-cancer and protective effect of the gut microbiome

Manipulation of the microbiota can have a protective effect against CRC due to the production of short-chain fatty acids (SCFAs), production of anti-oxidant enzymes, antiproliferative activity, and toxin-producing pathogens inhibition [16]. SCFAs and secondary bile acids (BAs) are bacterial metabolites, which, at high levels, have opposing protective effects on colon inflammation [23]. High-fat diets result in secondary BAs, which are risk factors for colonic cancer while primary BAs are vital in cholesterol metabolism, lipid digestion, and host-microbe interaction, whereas, higher dietary ﬁber intake results in the increased production of the SCFAs, propionate, acetate, and butyrate, which provide protection during the early stages of tumorigenesis [15]. Butyrate has anti-cancer activity; the absence of butyrate-producing microorganisms could increase inﬂammation and tumorigenesis [13]. Although not studied, it is hypothesized that a high-ﬁber diet inhibits the harmful effects of a high-fat diet [23]. Dietary ﬁber constitutes non-digestible food components, from which the gut microbiota produces SCFA. Studies show that dietary ﬁber hinders the BAs reabsorption and lowers circulating BAs levels. However, a low intake of fewer than 15 grams per day of dietary ﬁber results in decreased SCFA production, reduces microbial diversity, and shifts gut microbial metabolism toward using dietary fat, which increases bile acid production and increases detrimental metabolites.

The presence of friendly commensals in the gut protects the host mucosal tissue, immunologically strengthening the host defense system and excluding opportunistic pathogens [16]. Gut microbiota synthesizes and secretes bacteriocins, which are narrow or broad-spectrum antimicrobial peptides synthesized ribosomally, and keep pathogens like C. difficile, Campylobacter jejuni, Salmonella spp., and Listeria monocytogenes in check.

Probiotic bacteria, such as Bifidobacterium spp. and Lactobacillus spp., deactivate microbial enzymes and may contribute to anticarcinogenic effects. Lactic acid bacteria (LAB) were found to slow carcinogenesis, and concentrations of LAB increase after the consumption of foods containing probiotics, especially Kefir and yogurt [38]. Probiotic LAB can decrease the activity of β-glucuronidase, azoreductase, and nitroreductase.

Commensal gut microflora like Lactobacilli and bifidobacteria produce large amounts of acetic acid and lactic acid, maintaining a low pH level in the gut, inhibiting the growth of pathogenic bacteria and yeasts. While the yeast dies easily in such conditions, pathogenic bacteria survive due to an active proton pump and the commensal flora is resistant to these acids. The pathogenic survival is, however, short-lived and they are gradually eradicated, as they cannot keep up with the ATP supply required for the energy of their proton pumps.

## Conclusions

Although studies confirm an association and correlation between microbiota dysbiosis and CRCs, as well as point out specific pathogens and their mechanism of action on tumorigenesis, more studies are required to confirm not only correlation but causation in larger population groups. Future research should include and even focus on mycobiome and virome on CRC development. Due to the diversity of the gut microbiome, there is a high possibility that the gain and loss of bacteria and their metabolic functions lead to CRC.
